# Non-HLA Antibodies in Hand Transplant Recipients Are Connected to Multiple Acute Rejection Episodes and Endothelial Activation

**DOI:** 10.3390/jcm11030833

**Published:** 2022-02-04

**Authors:** Dorota Sikorska, Dorota Kamińska, Rusan Catar, Mirosław Banasik, Harald Heidecke, Kai Schulze-Forster, Katarzyna Korybalska, Rafał Rutkowski, Joanna Łuczak, Jerzy Jabłecki, Andrzej Oko, Przemysław Daroszewski, Mariusz Kusztal, Włodzimierz Samborski

**Affiliations:** 1 Department of Rheumatology, Rehabilitation and Internal Medicine, Poznan University of Medical Sciences, 61-545 Poznan, Poland; dyrektor@orsk.pl (P.D.); samborskiw@o2.pl (W.S.); 2 Department of Nephrology and Transplantation Medicine, Wroclaw Medical University, 50-556 Wroclaw, Poland; dorotakaminska@interia.pl (D.K.); m.banasik@interia.pl (M.B.); mariok@o2.pl (M.K.); 3 Department of Nephrology and Internal Intensive Care Medicine, Charité—Universitätsmedizin Berlin, Corporate Member of Freie Universität Berlin and Humboldt-Universität zu Berlin, 10117 Berlin, Germany; rusan.catar@charite.de; 4 CellTrend GmbH, 14943 Luckenwalde, Germany; heidecke@celltrend.de (H.H.); schufo@celltrend.de (K.S.-F.); 5 Department of Pathophysiology, Poznan University of Medical Sciences, 60-806 Poznan, Poland; koryb@ump.edu.pl (K.K.); rrutkowski@ump.edu.pl (R.R.); jgrzelczak@ump.edu.pl (J.Ł.); 6 Subdepartment of Replantation of Limbs, St. Hedwig of Silesia Hospital, 55-100 Trzebnica, Poland; jerzy.jablecki@interia.pl; 7 Deptartment of Health Science, Opole Medical School, 45-057 Opole, Poland; 8 Department of Nephrology, Transplantology and Internal Medicine, Poznan University of Medical Sciences, 60-355 Poznan, Poland; aoko@ump.edu.pl

**Keywords:** hand transplantation, non-HLA antibodies, rejection, vasculopathy, vascularized composite allotransplantation

## Abstract

The role of anti-HLA antibodies in transplant rejection is well-known but the injury associated with non-HLA antibodies is now widely discussed. The aim of our study was to investigate a role of non-HLA antibodies in hand allografts rejection. The study was performed on six patients after hand transplantation. The control group consisted of: 12 kidney transplant recipients and 12 healthy volunteers. The following non-HLA antibodies were tested: antibody against angiotensin II type 1 receptor (AT1R-Ab), antibody against endothelin-1 type-A-receptor (ETAR-Ab), antibody against protease-activated receptor 1 (PAR-1-Ab) and anti-VEGF-A antibody (VEGF-A-Ab). Chosen proinflammatory cytokines (Il-1, IL-6, IFNγ) were used to evaluate the post-transplant humoral response. Laboratory markers of endothelial activation (VEGF, sICAM, vWF) were used to assess potential vasculopathy. The patient with the highest number of acute rejections had both positive non-HLA antibodies: AT1R-Ab and ETAR-Ab. The same patient had the highest VEGF-A-Ab and very high PAR1-Ab. All patients after hand transplantation had high levels of laboratory markers of endothelial activation. The existence of non-HLA antibodies together with multiple acute rejections observed in patient after hand transplantation should stimulate to look for potential role of non-HLA antibodies in humoral injury in vascular composite allotransplantation.

## 1. Introduction

The results in vascularized composite allotransplantation (VCA) are promising but acute rejection rates are higher than in solid organs recipients, making it a clinically significant problem in VCA [1,2]. Furthermore, an increase in frequency of acute rejection episodes is probably correlated with chronic rejection [3]. In VCA, chronic rejection was initially felt to be less common. However chronic rejection, including vasculopathy, were also reported in more long-term VCA [4,5], chronic changes such as dermal capillary thromboses with C4d deposits in cutaneous capillaries have been seen [6]. Thus, it seems that in VCA, both acute and chronic rejection is a clinically significant problem and the development of graft vasculopathy may play a role in the long-term deterioration of hand allografts [7,8]. 

Much of the data on rejection and immunosuppression in VCA have been extrapolated from that of solid organ transplantation, which was precisely described in the literature data [7,8]. Although the pathologic changes somewhat differ according to the organ considered, a frequent feature of chronic rejection is injury of blood vessels, which may lead to graft vasculopathy. This vasculopathy is thought to be caused by low-grade inflammation-induced endothelial damage and chronic remodeling of smooth muscle cells in an attempt to heal this damage. This induces neointimal thickening and excess deposit of perivascular collagen. Progressive narrowing of the arterial lumen result in ischemic changes, graft fibrosis, dysfunction, and eventually, graft loss [7,8]. These vasculopathy patterns seem to reflect the microangiopathy observed in patients with active systemic sclerosis [9].

In solid organ transplantation, vasculopathy in allograft was traditionally associated with cell-mediated rejection, but the humoral involvement (antibody-mediated rejection) is increasingly recognized to be part of this process [10]. Many studies have shown that circulating donor specific antibodies against human leukocyte antigen (HLA) are linked with solid organ transplant rejection [11,12]. However, the process of humoral allograft rejection can also proceed in the absence of anti-HLA antibodies. There is an increasing amount of evidence suggesting the influence of non-HLA antibodies may induce allograft vasculopathy and injury, in both acute and chronic rejection [13,14,15,16,17]. Non-HLA antibodies may function as complement and non-complement fixing antibodies playing a role in acute and chronic damage in solid organ transplantation [18]. 

Mechanisms of VCA rejection have not yet been fully characterized. Probably the most rejection episodes are being cell-mediated (T cells have been implicated in acute rejection of skin allografts) [19]. However, cases of humoral anti-HLA antibody-mediated rejection in face- or hand-transplanted patients have also been reported (especially that it is difficult to find full compatibility in HLA antigens between the donor and the recipient, due to the small number of donors) [20,21]. Immunological factors involved in rejection of VCA probably are similar to those seen in the solid organ transplants [7,8] and VCA rejections can also occur in the absence of anti-HLA antibodies [22]. However, the role of non-HLA antibodies in VCA rejection has not yet been investigated. 

In our earlier study we found that hand transplant recipients present advanced microvascular abnormalities in nailfold capillaroscopic pattern connected with elevated levels of vascular endothelial growth factor (VEGF) [23]. These abnormalities resemble those seen in patients with active systemic sclerosis, characterized by inflammation and a progressive fibrosis affecting the skin [9]. Earlier, some authors concluded that patients after hand or face transplantation can develop chronic rejection in VCA with striking similarities to alterations seen in certain autoimmune cutaneous disorders, such as systemic sclerosis [24,25]. In systemic sclerosis non-HLA autoantibodies contribute to disease pathogenesis and may serve as biomarkers for risk assessment of disease progression [26]. Thus, we tentatively suggest that non-HLA antibodies may also be a part of high-risk immunologic profile capable of triggering a stronger alloimmune response and vasculopathy in VCA [27]. The fact that vasculopathy appears to be more pronounced in hand transplant patients than in solid-organ recipients may be because the skin is a very immunogenic tissue and the target for rejection-associated inflammation [28]. In the previous article we described the presence of non-HLA antibodies in combination with the occurrence of multiple rejection episodes found in one patient after bilateral hand transplantation [22]. To the best of our knowledge, there are only limited data available addressing the role of non-HLA immunity in VCA recipients. This prompted us to further investigation into a possible role of non-HLA antibodies and humoral immunity in hand allografts rejection.

## 2. Materials and Methods

### 2.1. Study Group

The study was performed on six patients, 2–11 years (mean 9 ± 5 years) after forearm (*n* = 5) or arm (*n* = 1) transplantation. The patients lost their limbs as a result of trauma and were in a good health before transplantation, without any co-morbidities. Before transplantation the serological HLA typing in donor and recipient was performed. Pre-transplant screening for donor specific antibodies carried out by cytotoxic crossmatch test was negative in all patients. All the recipients received immunosuppressive therapy: basiliximab in induction therapy, and tacrolimus (through levels 5–7 ng/mL), mycophenolate mofetil (1000–2000 mg daily) and prednisone (5 mg daily) as maintenance therapy [29]. After transplantation, the patients were routinely examined for allograft condition. There are no standard rules when a protocol biopsy should be performed. In Poland, protocol biopsies are performed every month during the first 3 months after transplantation and then yearly for the first 3 years. Later additionally biopsy-for-cause are performed in case of any rejection-indicating symptoms like skin erythema, limb swelling, deterioration of transplant function. All of the hand transplant recipients experienced at least one episode of acute rejection—most of the rejections occurred in the first year after transplantation. All the diagnosis of rejection was based on Banff classification of skin biopsies [30]. During the study all patients were in a stable clinical condition with no evidence of acute allograft rejection.

### 2.2. Control Groups

As a control group, the age- and sex-matched group of 12 stable kidney transplant recipients were selected. Pre-transplant screening for donor specific antibodies carried out by cytotoxic cross-match test was negative in all patients. The HLA typing results for kidney recipients and donors are presented in Supplementary Table S1. All received the same triple immunosuppression (tacrolimus with through levels about 5–6 ng/mL + mycophenolate mofetil (1000 mg/day) + prednisone (5 mg/d)) for a comparable period of time (10 ± 5 years) as hand transplant recipients. All the patients had (as an indication for kidney transplantation) primary kidney disease without co-morbidities. They also had a good function of the transplanted kidney, without any features (laboratory and imaging) of the rejection process—neither in the past nor at the time of the study. As an additional control group, 12 healthy volunteers of a similar age (mean 35 ± 5 years) were recruited from the general population.

### 2.3. Experimental Section

The study protocol conformed to the ethical guidelines of the 2000 Declaration of Helsinki and it was approved by the Poznan University Ethics Committee (No. 16/18). Written informed consent was obtained from each participant.

Basic demographic and medical data were collected from all patients during routine, periodic follow-up visits. To the microcirculation assessment, nailfold videocapillaroscopy was also performed in each patient. Nailfold videocapillaroscopy was performed by the same experienced operator (DS) using the CapillaryScope 200 MEDL4N microscope (Dino-Lite; Europe, Almere, The Netherlands)—as we described in our earlier study [23].

During this follow-up visit, an additional blood sample was also collected from each patient for additional laboratory determinations. 

### 2.4. Biochemical Analyses

The following antibodies were selected from non-HLA antibodies: antibody against angiotensin II type 1 receptor (AT1R-Ab), antibody against endothelin-1 type-A-receptor (ETAR-Ab), antibody against protease-activated receptor 1 (PAR-1-Ab), and anti-VEGF-A antibody (VEGF-A-Ab). The antibody levels were measured as previously described [31]. Non-HLA-AT1R-Ab and ETAR-Ab were considered positive when the result was above 10 U/mL.

Basal proinflammatory cytokines were used to evaluate the post-transplant humoral response: interferon-gamma (IFN-γ), interleukin-1-beta (IL-1β), interleukin-6 (IL-6). Basic markers were also used to assess endothelial activation and potential vasculopathy: vascular endothelial growth factor (VEGF), soluble intercellular adhesion molecule-1 (sICAM-1), von Willebrand factor (vWf). The immunoassay was performed as per manufacturer’s instructions. Concentration of hsIL-6 was determined by immunoassays from BioVendor (Brno, Czechia), concentration of IFN-γ and hsIL-1β were determined using immunoassays kits from R&D Systems (Minneapolis, MN, USA), whereas concentration of VEGF, sICAM-1 and vWf were measured using DuoSets from R&D Systems (Minneapolis, MN, USA). The sensitivity of the assays was: 0.32 pg/mL for hsIL-6, 1.28 pg/mL for IFN-γ, 0.033 pg/mL for hsIL-1beta, 12.2 pg/mL for VEGF, 17.5 pg/mL for sICAM-1, 0.10 ng/mL for vWf.

Only in hand transplant recipients we assessed the presence of anti-HLA antibodies with flow-PRA method (One Lambda/Thermo Fisher Inc., Canoga Park, CA, USA). Using solid-phase immunoassay technology (Luminex) we retested the presence of donor-specific anti- HLA antibodies (DSA) in all patients after hand transplantation. We did not find DSA.

### 2.5. Statistical Methods

Since the number of patients was too small to ascertain normality of the data distribution, the data were presented as medians (and interquartile ranges) and non-parametric tests were applied for statistical analysis. The data were analyzed with the Mann–Whitney or Kruskal–Wallis ANOVA tests, as required. Categorized data were analyzed with the χ2 test. The differences were considered significant at *p* < 0.05. All statistical analyses were performed using the Statistica 13.0 software (StatSoft Polska, Krakow, Poland).

## 3. Results

### 3.1. Patient with Positive Result of AT1R-Ab and also ETAR-Ab and the Highest Number of Rejections

We noticed that only one patient (patient T) developed positive result of both non-HLA antibodies: AT1R-Ab and also ETAR-Ab. Patient T developed no anti-HLA neither class 1 not class 2. We noticed in patient T the highest number of rejection’s episodes. Patient T had also the highest level of VEGF-A-Ab (5.56 U/mL) and one of the highest PAR1-Ab (7.91 U/mL) but also VEGF (631 pg/mL). The vWF (3.03 ng/mL) was also the highest (Table 1). It should be emphasized that the observation of the patient T is the shortest (2 years) in comparison to others (median 9 years).

### 3.2. The Analysis of the Group of Patient after Hand Transplantation

All hand transplant recipients presented more acute rejection episodes in comparison to patients after kidney transplantation (who presented no rejection episodes). In our studied group, three hand transplant recipients developed one or two acute rejections, and three were diagnosed to have multiple rejection episodes (5–6 episodes). Due to the small size of the patient group, it is not possible to perform a reliable statistical analysis, comparing patients with single (*n* = 3) and recurrent (*n* = 3) acute rejections- there seem to be no significant differences. It should be emphasized that all patients developed changes in microcirculation. One patient had anti-HLA in class 1 and the other had anti-HLA in class 2.

### 3.3. The Comparison of Patients after Hand and Kidney Transplantation

Patients after hand transplantation developed vascular changes in microcirculation which were found in nailfold videocapillaroscopy—what we described exactly in the previous study [23]. Vascular changes in hand and kidney transplant recipients seem to be indirectly confirmed also by laboratory markers of endothelial activation: VEGF, SICAM-1, and vWF levels were significantly higher in patients after hand transplantation in comparison to healthy control group. Additionally, VEGF levels were even higher after hand transplantation than after kidney transplantation (Table 2). 

There were no statistically significant differences in the concentrations of non-HLA antibodies between hand—and kidney—allograft recipients when they were analyzed in the whole group. Surprisingly, comparable non-HLA antibodies levels were also observed in the healthy control group (Table 3).

Simultaneously, in hand transplant recipients no higher levels of serum inflammatory markers (which could indicate the activation of endothelium) were observed (Table 4).

## 4. Discussion

The main aim of our study was to evaluate the role of non-HLA antibodies in hand transplant rejection. On the basis of the obtained results, we think that multiple episodes of acute rejection in hand trans-plant recipients could be related to higher levels of non-HLA antibodies: AT1R-Ab and also ETAR-Ab accompanying by the highest levels of endothelial activation markers (VEGF-A-Ab and PAR1-Ab). In our analysis patient (T) with positive AT1R-Ab but additionally positive ETAR-Ab, they had higher VEGF and also the highest vWF. This patient developed the highest (six) number of acute rejection episodes. The same patient had the highest VEGF-A-Ab and very high PAR1-Ab. There was no statistical significance probably because of few cases. Such observation should stimulate the discussion about the probable role of non-HLA antibodies in immunological injury of vessels which consequently may lead to vasculopathy. It should be emphasized that Patient T was observed for only 2 years and should be very carefully controlled in the next years of observation. It means that meticulous control is necessary but also maintaining of adequate immunosuppression to avoid potential immunological injury. 

In our recent studies we described the role of AT1 receptors but also ETA receptors in different renal compartments [32,33,34,35]. The stimulated injury described by authors as vascular changes in microcirculation [23] creates the dangerous opportunity for further damage of the transplant. Endothelium is located in a critical place between interstitial and intravascular compartments. Transplantologists should reflect endothelial activation and dysfunction in the pathogenesis of transplant injury [36].

There were no statistically significant difference in the concentrations of non-HLA antibodies between patients after hand transplantation (with episodes of acute and chronic rejection) and stable patients after kidney transplantation (without any features of the rejection process—neither in the past nor at the time of the study). Additionally, comparable values were observed in the healthy control group. Such results may be explained with the low number of patients and only one patient after hand transplantation, and who had a high activity of non-HLA antibodies, presented a positive result of AT1R-Ab and additionally positive ETAR-Ab. The role of non-HLA antibodies in VCA rejection has not yet been deeply investigated. To the best of our knowledge, our earlier study, which revealed the presence of non-HLA antibodies (in the absence of anti-HLA antibodies) in a hand transplant patient who developed six episodes of acute rejection of both hands (unlike patients with isolated episodes of acute rejections who were negative for non-HLA antibodies), is the only such analysis [22]. However, non-HLA antibodies (as alloantibodies or autoantibodies) occur in various contexts of solid organ transplantation, causing vasculopathy in allograft—which has been described in detail in numerous reviews [37,38,39,40]. Non-HLA antibodies are also involved in pathophysiology of autoimmune vascular disease—such as systemic sclerosis [26,41]. The involvement of non-HLA antibodies in hand allograft vasculopathy also may not be excluded in our observation. 

We have previously reported that stable hand transplant recipients exhibit persistent immune activation with rejection-related gene expression pattern [42]. Our present study showed that in hand transplant recipients no higher levels of serum inflammatory markers (IFN-γ, IL-I, and IL-6) were observed. Indeed, patients after kidney transplantation had higher concentrations of IL-6 than hand transplant recipients and control group (*p* = 0.002 and *p* = 0.005, respectively)- which may be caused by many factors [43,44,45] and is not the subject of this study. The role of the humoral response in rejection of solid organ transplantation has been well described [46,47,48] and much of the data on rejection in VCA have been extrapolated from that of solid organ transplantation [7,28,49]. It is also thought that in systemic sclerosis, humoral immune abnormalities link the vasculopathy [50,51,52]. However, the significance of humoral response to VCA vasculopathy and rejection is still unclear. To the best of our knowledge, no full analysis of proinflammatory cytokine concentrations and their role in VCA has been performed so far. The results of our study may indicate the lack of significant contribution of humoral response in the process of chronic rejection in hand transplant recipients.

At the same time, patients after hand transplantation showed advanced microvascular abnormalities in nailfold capillaroscopic pattern connected with elevated levels of VEGF, which may suggest vasculopathies—as we described in our earlier article [23]. In this study, vascular changes in hand transplant recipients seem to be indirectly confirmed also by other laboratory markers: sICAM and vWf. Earlier studies indicated that VEGF [53], sICAM [54], and vWf [55] concentrations may be markers of solid organ rejection. The role of these endothelial markers as predictors of vascular changes in systemic sclerosis has also been confirmed in numerous studies [56,57,58]. The above-mentioned endothelial markers reflect vascular changes, both in transplantation and in autoimmune diseases. Therefore, elevated endothelial markers may confirm vasculopathy also in our group after hand transplantation. 

Based on the results of our study, it can be assumed that non-HLA antibodies should be analyzed to control the humoral response, but also avoid vasculopathy and the process of chronic rejection in hand transplant recipients. The mechanisms underlying VCA vasculopathy are not clear. They may include both immunological and non-immunological (mechanical, cytotoxic, and thermal) insults that lead to endothelial cell damage and vessel remodeling [7,8,59,60]. An important factor responsible for the vascular changes in systemic sclerosis is tissue hypoxia. Tissue hypoxia increases the production of hypoxia inducible factor (HIF) and HIF itself increases the level of vascular endothelial growth factor (VEGF). Thus, the tissues hypoxia is a stimulus for new blood vessels formation. However, neoangiogenesis occurs with the formation of abnormal vessels, what is visible in the nailfold capillary examination [61,62]. The capillaroscopic pattern in systemic sclerosis is very similar to that observed in patients after hand transplantation. It is possible that also in patients after hand transplantation, hypoxia (ischemia-reperfusion injury) is the factor responsible for vasculopathy. Nevertheless, this requires further analysis.

The responses to HLA on the allograft have traditionally been considered as the main mechanism of acute solid organ rejection [63]. However, the influence of non-HLA antibodies is increasingly recognized [64]. In our studied group episodes of acute rejection were observed only in patients after hand transplantation (three patients—single rejections—three recurrent ones). Unfortunately, the insufficient number of patients and the analysis in a stable period between episodes of acute rejection prevented a complete, reliable, comparative statistical analysis. However, the patient with the highest number of acute rejections (while being the shortest post-transplant) had the highest concentrations of all non-HLA antibodies, while showing higher VEGF and vWF values. Perhaps, therefore, non-HLA antibodies play some role in the process of acute rejection, which seems to be confirmed also by our earlier report on another patient with recurrent acute rejection [22]. However, there are no other analyses on this subject in the literature. 

Our study has significant limitations. First of all, it is a small study group (there are no more patients after hand transplants in our country). In addition, this is a cross-sectional study in which patients were assessed at only one time point—there is no pre-transplant analysis of patients, which could be especially useful to assess whether the presence of non-HLA antibodies was due to auto- or allo-immunization. Unfortunately, our study also did not evaluate the mechanisms of cellular rejection, which may be one of the important elements of VCA rejection. Thus, more research is needed on the role of non-HLA antibodies in VCA rejection, especially as new therapeutic possibilities are emerging, consisting of blocking specific non-HLA antibodies, e.g., AT1R antibodies [65].

## 5. Conclusions

Non-HLA antibodies in hand transplant recipients are connected to multiple acute rejection episodes and endothelial activation. The existence of non-HLA antibodies (anti-AT1R and anti-ETAR) together with multiple acute rejections observed in patient after hand transplantation should stimulate to careful care, but also upcoming research should look for the potential role of non-HLA antibodies in antibody-mediated injury.

## Figures and Tables

**Table 1:** Detailed characteristics of hand transplant recipients.

Patient (the First Letter of the Surname)	(H)	(O)	(W)	(B)	(S)	(T)
No of acute rejections	1	1	2	5	5	6
Age (years)	33	45	64	38	38	33
Time after transplantation (years)	3	11	8	9	9	2
Changes in microcirculation in capillaroscopy (yes/no)	Y	Y	Y	Y	Y	Y
No of HLA mismatch	6	5	4	3	5	6
Donor HLA	A-1,2, B-8,57, DR-3,7	A-31,32, B-35,39, DR-11,16	A-2, B-7,39, DR-13,15	A-26, B-40,56, DR-1,13	A-1,3, B-7,8, DR-3,11	A-1,68 B-7,8 DR-13,15
Recipient HLA	A-24,25, B-27, DR-1,4	A-24, B-18,40, DR-1,11	A-1,2 B-8,15, DR-3,4	A-24,26, B-13,38, DR-7,13	A-2,3, B-35,44, DR-13,16	A-2,3 B-27,51 DR-1,8
Anti-HLA cl. I (yes/no)	N	N	Y	N	N	N
Anti-HLA cl.II (yes/no)	N	N	N	N	Y	N
AT1R-Ab (U/mL)	6.25	9.98	8.95	5.88	8.24	11.73
ETAR-Ab (U/mL)	6.47	9.21	7.64	6.76	9.15	11.21
PAR1-Ab (U/mL)	3.62	4.02	8.01	1.24	2.62	7.91
VEGF-A-Ab (U/mL)	2.37	3.92	3.67	2.33	2.87	5.56
VEGF (pg/mL)	414.4	564.2	670.2	442.8	319.8	631.2
sICAM-1 (ng/mL)	108.19	145.68	183.29	167.85	254.80	164.98
vWF (ng/mL)	0.75	1.56	1.40	1.74	1.49	3.08
IFN-gamma (pg/mL)	2.00	23.64	6.71	2.29	1.96	1.87
hs Il-1 (pg/mL)	0.37	1.08	0.51	0.32	0.64	0.62
hs Il-6 (pg/mL)	3.01	2.73	3.45	2.54	5.15	3.26

HLA—human leukocyte antigen, AT1R-Ab—antibody against angiotensin II type 1 receptor, ETAR-Ab—antibody against endothelin-1 type-A-receptor, PAR-1-Ab—antibody against protease-activated receptor 1, VEGF-A-Ab—anti-vascular endothelial growth factor-A antibody, VEGF—vascular endothelial growth factor, sICAM-1—soluble intercellular adhesion molecule-1, vWf—von Willebrand factor, IFN-γ—interferon-gamma, IL-1β—interleukin-1-beta, IL-6—interleukin-6.

**Table 2:** Markers of endothelial activation in study groups.

	Patients after Hand Transplantation (*n* = 6)	Patients after Kidney Transplantation (*n* = 12)	Healthy Control Group (*n* = 12)	*p*-Value (ANOVA Test)
VEGF (pg/mL) *	503.5 (414.4–631.2)	194.0 (123.3–259.5)	50.0 (17.5–90.8)	<0.001 ^1^
sICAM-1 (ng/mL)	166.4 (145.7–183.3)	135.7 (122.4–161.6)	92.8 (69.2–114.6)	0.003 ^2^
vWf (ng/mL)	1.5 (1.4–1.7)	1.5 (1.2–1.8)	0.7 (0.5–0.9)	<0.001 ^3^

Data are presented as a median (interquartile range). VEGF—vascular endothelial growth factor, sICAM-1—soluble intercellular adhesion molecule-1, vWf—von Willebrand factor. * VEGF data have been previously published in our earlier study [22]. ^1^ Hand vs. kidney: *p* = 0.001, hand vs. healthy: *p* < 0.001, kidney vs. healthy: *p* < 0.001 (Mann–Whitney test). ^2^ Hand vs. kidney: *p* = 0.174, hand vs. healthy: *p* = 0.013, kidney vs. healthy: *p* = 0.003 (Mann–Whitney test). ^3^ Hand vs. kidney: *p* = 0.743, hand vs. healthy: *p* = 0.010, kidney vs. healthy: *p* < 0.001 (Mann–Whitney test).

**Table 3:** Non-HLA antibodies levels in subgroups.

	Patients after Hand Transplantation (*n* = 6)	Patients after Kidney Transplantation (*n* = 12)	Healthy Control Group (*n* = 12)	*p*-Value (ANOVA Test)
AT1R-Ab (U/mL)	8.60 (6.25–9.98)	9.70 (8.60–11.66)	9.68 (8.26–11.83)	0.401
ETAR-Ab (U/mL)	8.40 (6.76–9.21)	9.67 (8.04–13.36)	10.07 (9.19–13.09)	0.185
PAR-1-Ab (U/mL)	4.57 (2.62–7.90)	5.37 (2.55–10.77)	2.40 (1.99–4.70)	0.203
VEGF-A-Ab (U/mL)	3.26 (2.37–3.92)	4.65 (3.01–5.76)	3.73 (2.69–7.02)	0.606

Data are presented as a median (interquartile range). AT1R-Ab—antibody against angiotensin II type 1 receptor, ETAR-Ab—antibody against endothelin-1 type-A-receptor, PAR-1-Ab—antibody against protease-activated receptor 1, VEGF-A-Ab—anti-vascular endothelial growth factor-A antibody. Hand vs. healthy, kidney vs. healthy, and hand vs. kidney- all NS (Mann–Whitney test).

**Table 4:** Pro-inflammatory cytokines levels in subgroups.

	Patients after Hand Transplantation (*n* = 6)	Patients after Kidney Transplantation (*n* = 12)	Healthy Control Group (*n* = 12)	*p*-Value (ANOVA Test)
IFN-γ (pg/mL)	2.14 (1.96–6.71)	1.95 (1.75–3.26)	1.86 (1.80–2.44)	0.295
IL-1β (pg/mL)	0.56 (0.37–0.64)	0.40 (0.33–0.45)	0.48 (0.33–0.58)	0.287
IL-6 (pg/mL)	3.13 (2.72–3.44)	6.20 (4.91–7.03) ^#^	2.60 (2.34–4.20)	0.002

Data are presented as a median (interquartile range). IFN-γ—interferon-gamma, IL-1β—interleukin-1-beta, IL-6—interleukin-6. Hand vs. kidney and hand vs. healthy-all ns; ^#^ kidney vs. healthy: *p* < 0.01 (Mann–Whitney test).

## Data Availability

Not applicable.

## Supplementary Materials

The following are available online at https://www.mdpi.com/article/10.3390/jcm11030833/s1, Table S1: HLA typing results for kidney recipients and donors.
