# Supplementary material for: Non-HLA Antibodies in Hand Transplant Recipients Are Connected to Multiple Acute Rejection Episodes and Endothelial Activation
Source: J Clin Med. 2022 Feb 4;11(3):833. doi: 10.3390/jcm11030833 (PMC8837026; doi:10.3390/jcm11030833)
Supplement: Supplementary file 1 [file jcm-11-00833-s001.zip › jcm-1578340-supplementary.pdf]

Supplementary Table S1. HLA typing results for kidney recipients and donors.

Patient Number	Donor HLA	Recipient HLA	AT1R-Ab Units/mL	ETAR-Ab Units/mL	PAR1-Ab Units/mL	VEGF-A-Ab Units/mL
1	A 3,30 B 7,35, DR 11,15	A2,25, B 18,40, DR 11,15	7,3	6,6	1,3	1,2
2	A 1,2, B 37,44, DR 11,15	A 24,68 B 38,44, DR 4,11	9,7	7,7	9,0	7,4
3	A3,3, B 7,56, DR 11,13	A 2,31, B 15,39, DR 12,13	9,8	10,7	5,9	4,8
4	A 3,30, B 7,35, DR 11,15	A 1,26, B 35,38, DR 11,15	17,9	15,0	4,9	5,4
5	NA	NA	12,4	13,2	20,6	6,3
6	A 2,3 B 5,6 DR 1,6	A 1,2 B 15,37, DR 4,6	9,6	10,8	2,5	3,1
7	A 2,32, B 13,18, DR 9,13	A 1,33, B 14,37, DR 1,10	7,6	8,0	1,8	2,9
8	NA	NA	9,7	8,4	2,6	3,8
9	A 3,10, B 5,6, DR 1,6	A 2,2 B 14,15, DR 1,6	12,2	15,4	13,7	4,5
10	A 3,26, B 8,35, DR 12,17	A 1,21, B 8,41, DR 11, 17	9,0	8,6	4,1	2,8
11	A 1,2, B 18,44, DR 7,16	A 1,2, B 44,50, DR 1,7	11,1	13,5	12,6	5,5
12	NA	NA	8,3	8,1	7,6	6,1

NA—No data for patients who had a transplant before 2006, no database in the system.
